# Case Report: Endoscopic geometric remodeling for angulation-type efferent loop stenosis after pancreaticoduodenectomy

**DOI:** 10.3389/fmed.2026.1773119

**Published:** 2026-06-18

**Authors:** Donghai Wu, Shihua Ding, Na Li, Weitao Wu, Jingbo Yang, Jiahuang Huang

**Affiliations:** The First Affiliated Hospital of Shenzhen University, Shenzhen Second People’s Hospital, Shenzhen, China

**Keywords:** case report, covered self-expanding metal stent, delayed gastric emptying, efferent loop stenosis, endoscopic submucosal dissection, pancreaticoduodenectomy, traction clips

## Abstract

**Background:**

Efferent loop stenosis (ELS) after pancreaticoduodenectomy (PD) with gastrojejunostomy reconstruction is an uncommon cause of delayed gastric emptying. Reoperation in the early postoperative period carries substantial risk. Endoscopic balloon dilation and stenting are often used for benign postoperative strictures, but when fixed angulation rather than a short concentric narrowing is the main problem, these approaches may be unsafe or ineffective.

**Case description:**

A 51-year-old woman with a more than 2-year history of intermittent epigastric fullness and pain underwent laparoscopic PD with Child reconstruction for an approximately 2 cm pancreatic head mass. Imaging suggested a pancreatic neuroendocrine tumor and histopathology confirmed a grade 2 pancreatic neuroendocrine tumor (Ki-67 about 5%) with negative margins. The early postoperative course was initially stable. On postoperative day 7, she developed upper abdominal distension and high-output bilious nasogastric drainage (800–1,100 mL/day), and oral intake could not be advanced. Conservative treatment with nasogastric decompression, bowel rest, intravenous fluids, proton-pump inhibitor therapy, and prokinetic agents was continued for five days but did not improve the symptoms. A barium meal examination showed delayed gastric emptying with impaired flow into the efferent limb. Upper endoscopy demonstrated viscous bile pooling in the stomach and a sharply angulated efferent loop at the gastrojejunostomy, without a discrete anastomotic ring stricture. These findings were consistent with ELS caused mainly by loop angulation. Because stable coaxial positioning of a balloon catheter across the kinked segment was considered difficult and potentially unsafe, we chose a combined strategy. Endoscopic submucosal dissection (ESD) was used to partially remove a saddle-shaped mucosal ridge at the efferent-loop entrance. Clips were applied for hemostasis and to provide traction, straightening the efferent loop and improving alignment with the gastric lumen. A 20 mm × 80 mm fully covered self-expanding metal stent (FCSEMS) was then placed to maintain patency. After the procedure, the patient’s abdominal distension improved, nasogastric drainage decreased, and the tube was removed on day 3. Oral intake was gradually resumed. At 1 month, a contrast study and repeat endoscopy showed good passage through a straightened efferent limb, and the FCSEMS was removed without complications. At 3 months, she reported good appetite, a weight gain of about 5 kg, and endoscopy showed a widely patent efferent loop without recurrent stenosis.

**Conclusion:**

ESD-assisted mucosal release with clip-based traction and temporary FCSEMS placement corrected angulation-type ELS early after PD in this patient. This endoscopic approach, which focuses on modifying the loop geometry rather than simple radial dilation, may be considered as an alternative to surgical revision in carefully selected cases where standard balloon dilation is not feasible.

## Introduction

Pancreaticoduodenectomy (PD) is widely used for malignant and selected benign lesions of the pancreatic head and periampullary region. Despite improvements in surgical techniques and perioperative care, the procedure is still associated with considerable morbidity. Postoperative pancreatic fistula and delayed gastric emptying (DGE) remain among the most frequent complications (1).

Efferent loop stenosis (ELS) after gastrojejunostomy reconstruction is a relatively rare but clinically important cause of postoperative obstruction and DGE. Patients may present with persistent nausea, vomiting, abdominal distension and poor oral intake, which can lead to prolonged hospitalization and malnutrition. Early reoperation in this context is often difficult because of adhesions, tissue fragility and the risk of further complications (1).

Endoscopic balloon dilation (EBD) and temporary stenting are well-established treatments for benign postsurgical strictures in the upper gastrointestinal tract, including anastomotic strictures after PD and hepaticojejunostomy (2–4). These methods rely on achieving a stable, coaxial position of the balloon or stent across a short, concentric narrowing and applying controlled radial force. When the main mechanism is fixed angulation or kinking of the efferent limb, rather than a fibrotic stricture, conventional dilation may not address the geometric problem and may even be unsafe if stable access cannot be obtained (2, 4).

We report a case of early postoperative, angulation-type ELS after PD that was treated using a combined endoscopic approach. Limited ESD was performed to release a saddle-shaped mucosal ridge at the efferent-loop entrance, clips were used to provide traction and straighten the efferent limb, and a temporary fully covered self-expanding metal stent (FCSEMS) was placed to maintain patency. We describe the clinical course and discuss how this strategy may be applied in similar situations.

## Case description

This case report was prepared in accordance with the CARE guidelines. The clinical timeline is summarized in Table 1.

**Table 1:** Timeline of the episode of care.

Time point	Key findings	Management/outcomes
Preoperative	More than 2-year history of intermittent symptoms; imaging suggested pancreatic neuroendocrine tumor.	Planned for pancreaticoduodenectomy after multidisciplinary discussion.
Surgery (Day 0)	Laparoscopic pancreaticoduodenectomy with Child reconstruction; operative time 12 h; hand-sewn gastrojejunostomy to the posterior wall of the remnant stomach; pathology: grade 2 pancreatic neuroendocrine tumor (Ki-67 about 5%) with negative margins.	No intraoperative nasojejunal tube or feeding jejunostomy; uncomplicated intraoperative course.
Postoperative day 7	Upper abdominal distension and increased nasogastric drainage (bilious, 800–1,100 mL/day).	Oral intake attempts failed; delayed gastric emptying suspected.
Next 5 days	Persistent large-volume bilious drainage.	Conservative management including continuous nasogastric decompression, fluid/electrolyte replacement, proton-pump inhibitor therapy, and prokinetics (metoclopramide 10 mg IV q8 h; erythromycin 250 mg IV q12 h); early mobilization and postural changes.
Diagnostic assessment	Barium study and endoscopy suggested angulation-type efferent loop stenosis rather than a short fibrotic stricture; the afferent limb was patent.	Multidisciplinary review favored postoperative complication-related angulation; decision made to pursue endoscopic geometric remodeling rather than balloon dilation alone.
Endoscopic intervention	Saddle-shaped mucosal ridge and angulated efferent loop entrance.	Surgical revision was discussed as a fallback option; limited ESD-assisted mucosal release, clip-based traction to straighten the limb, and temporary fully covered SEMS (20 mm x 80 mm) were performed.
Post-intervention day 3	Symptoms improved.	Nasogastric tube removed; oral intake gradually resumed.
1 month	Contrast study and repeat endoscopy showed good passage.	Stent removed endoscopically without difficulty.
3 months	Good appetite; no recurrent stenosis; weight gain about 5 kg.	No late adverse events.

### Patient information and surgery

A 51-year-old woman was admitted with a more than 2-year history of intermittent epigastric fullness and pain. The symptoms had worsened in the 5 days before admission. She had no major comorbidities and no previous abdominal surgery. Contrast-enhanced computed tomography revealed an approximately 2 cm, well-demarcated mass in the pancreatic head, with imaging features suggestive of a pancreatic neuroendocrine tumor.

After multidisciplinary discussion, laparoscopic PD with Child reconstruction was performed; the operative time was 12 h. The reconstruction included pancreaticojejunostomy, hepaticojejunostomy, and antecolic gastrojejunostomy. The gastrojejunostomy was constructed on the posterior wall of the remnant stomach using a hand-sewn technique. No nasojejunal tube or feeding jejunostomy was placed intraoperatively. The operation proceeded without intraoperative complications.

Histopathology confirmed a grade 2 pancreatic neuroendocrine tumor with a Ki-67 index of about 5% and negative resection margins. These findings were consistent with the diagnosis and grading criteria described in current guidelines for pancreatic neuroendocrine tumors (5).

### Clinical findings and postoperative course

In the first postoperative week, the patient’s condition was stable. Postoperative imaging showed no clear evidence of pancreatic leakage or retrogastric collection. On postoperative day 7, she developed upper abdominal distension and increased nasogastric drainage. The drainage was bilious, with a daily volume of 800–1,100 mL. Attempts to advance oral intake failed because of recurrent symptoms.

Conservative measures were started and continued for five days. Management included continuous nasogastric decompression, nil per os, intravenous fluid and electrolyte replacement, proton-pump inhibitor therapy, and prokinetic agents (metoclopramide 10 mg intravenously every 8 h and erythromycin 250 mg intravenously every 12 h). Early mobilization and postural changes were encouraged.

**Figure 1:**
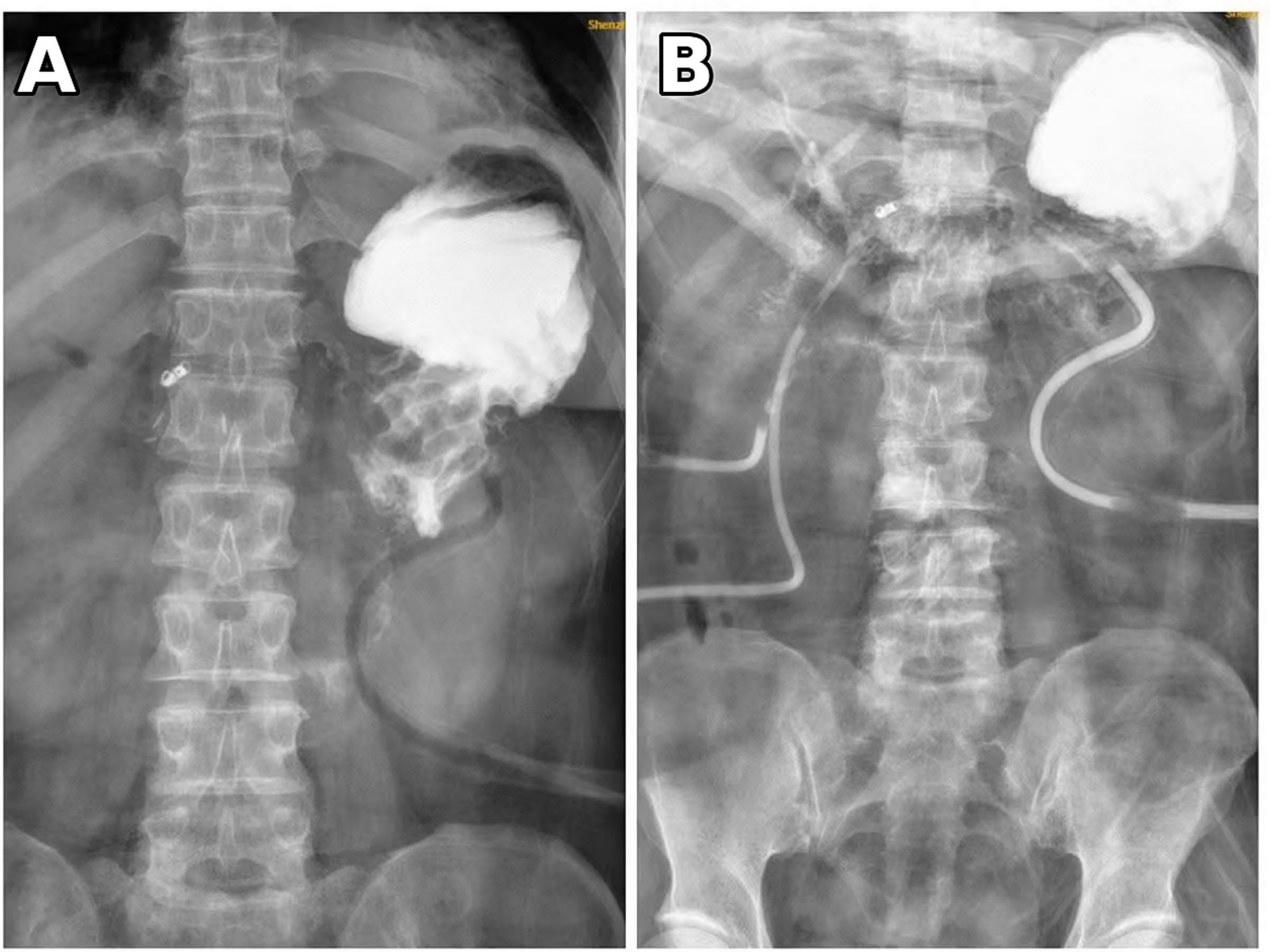
Pre-intervention contrast study. **(A)** Abdominal radiograph obtained 30 min after contrast instillation through the nasogastric tube, showing retention of contrast in the remnant stomach with limited downstream passage. **(B)** Radiograph obtained 3.5 h after contrast instillation, showing persistent gastric retention and delayed emptying into the efferent limb.

Despite these measures, large-volume bilious drainage persisted and oral intake could not be resumed. A barium meal examination showed delayed gastric emptying and impaired flow of contrast into the efferent limb of the gastrojejunostomy (Figure 1). These findings suggested clinically relevant postoperative DGE.

### Diagnostic assessment

Upper gastrointestinal endoscopy was performed to clarify the cause of DGE. Viscous bile was seen in the stomach. At the gastrojejunostomy, the efferent-loop entrance was sharply angulated and difficult to intubate. A transparent cap was needed to manipulate the endoscope tip into the efferent limb. The afferent limb was patent. There was no obvious anastomotic ring narrowing or ulceration. The overall impression was that of a kinked and probably adherent efferent loop rather than a short segment fibrotic stricture. After review of the postoperative endoscopic and imaging findings during multidisciplinary discussion, the efferent-limb angulation was considered more likely to be related to postoperative complication-associated local anatomical distortion or adhesion than to a definite technical fault during the initial reconstruction.

### Therapeutic intervention

EBD and temporary stent placement are commonly used for benign postoperative strictures in the upper gastrointestinal tract, including anastomotic strictures after hepatobiliary and pancreatic surgery (2–4). These techniques work best for short, concentric narrowing where a guidewire and balloon or stent can be passed in a stable, coaxial fashion.

In this patient, the efferent-loop entrance was sharply angulated and appeared fixed. Under endoscopic and fluoroscopic assessment, the geometry of the kink suggested that positioning a balloon catheter across the angulated segment would be technically challenging and might carry a risk of perforation or other injury. Furthermore, simple dilation alone was unlikely to correct the underlying angulation.

Before endoscopic treatment, surgical revision was discussed with the patient as a fallback option if endoscopic therapy failed or if procedure-related complications occurred. After discussion of the potential benefits and risks, the patient requested an attempt at endoscopic treatment first. For these reasons, we chose an approach aimed at changing the geometry of the efferent limb rather than only dilating a presumed stricture. The plan was to perform limited ESD to release the saddle-shaped mucosa at the efferent-loop entrance, use clips to provide traction and straighten the efferent limb, and then place a temporary FCSEMS to maintain patency during healing.

The procedure was carried out using a therapeutic gastroscope with a transparent cap. After careful inspection of the gastrojejunostomy, a saddle-shaped mucosal ridge at the efferent-loop entrance was identified. This mucosal structure seemed to contribute to the angulation and narrowing of the efferent lumen.

A submucosal injection containing normal saline, hyaluronate and indigo carmine was given beneath the saddle-shaped mucosa to lift the lesion. Using standard ESD techniques, a mucosal incision was made and a limited submucosal dissection was performed to partially resect the saddle-shaped mucosa. This released the mucosal bridge and helped to open the efferent-loop entrance.

Hemostatic clips were applied along the resection margin to secure hemostasis and reduce the exposed wound. Additional clips were then placed in a way that provided traction, pulling the efferent-loop entrance in a direction that straightened the loop and improved alignment with the gastric lumen. The new orientation was confirmed under endoscopy and fluoroscopy.

**Figure 2:**
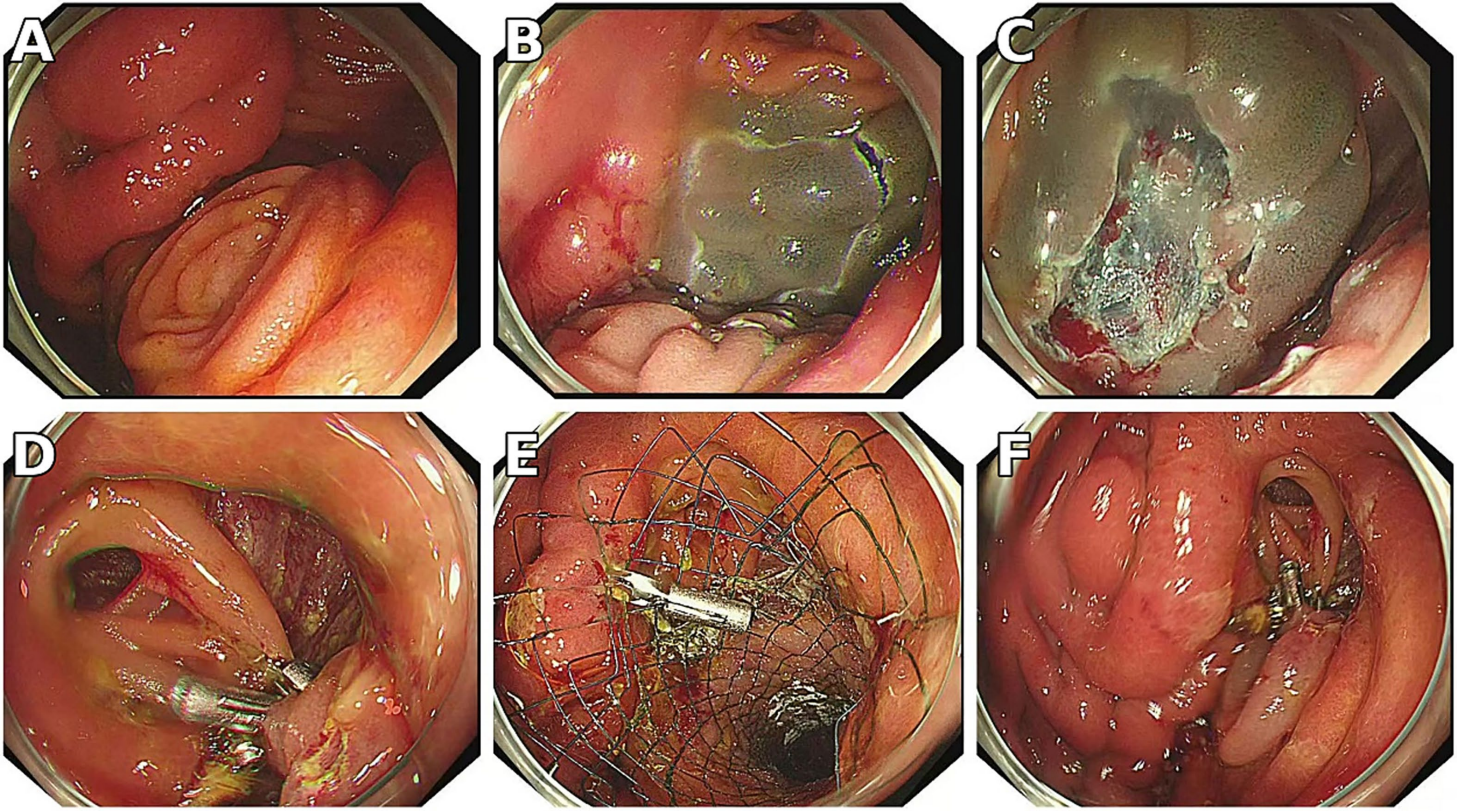
Endoscopic treatment of angulation-type efferent-loop stenosis. **(A)** Sharply angulated efferent-loop entrance at the gastrojejunostomy, with difficult access. **(B)** Submucosal injection beneath the saddle-shaped mucosal ridge. **(C)** Limited ESD of the ridge to release the mucosal component of the obstruction. **(D)** Clip placement at the resection margin and efferent-loop entrance to provide traction and improve alignment. **(E)** Deployment of a fully covered self-expanding metal stent across the corrected efferent limb. **(F)** Final endoscopic view showing improved alignment and luminal patency.

After the loop had been straightened, a 20 mm × 80 mm fully covered self-expanding metal stent (Niti-S™) was deployed within the efferent limb. The proximal end of the stent protruded slightly into the gastric side of the anastomosis, acting as an internal splint and maintaining luminal patency (Figure 2). Final endoscopic inspection showed a widely open efferent lumen, stable clip placement and no immediate complications.

### Follow-up and outcomes

Following the endoscopic procedure, the patient remained nil per os for 72 h and received intravenous nutritional support. Her abdominal distension gradually improved, and the volume of nasogastric drainage decreased. The nasogastric tube was removed on day 3 after the endoscopic intervention. A liquid diet was then started and gradually advanced to a semi-liquid and soft diet, which she tolerated well.

**Figure 3:**
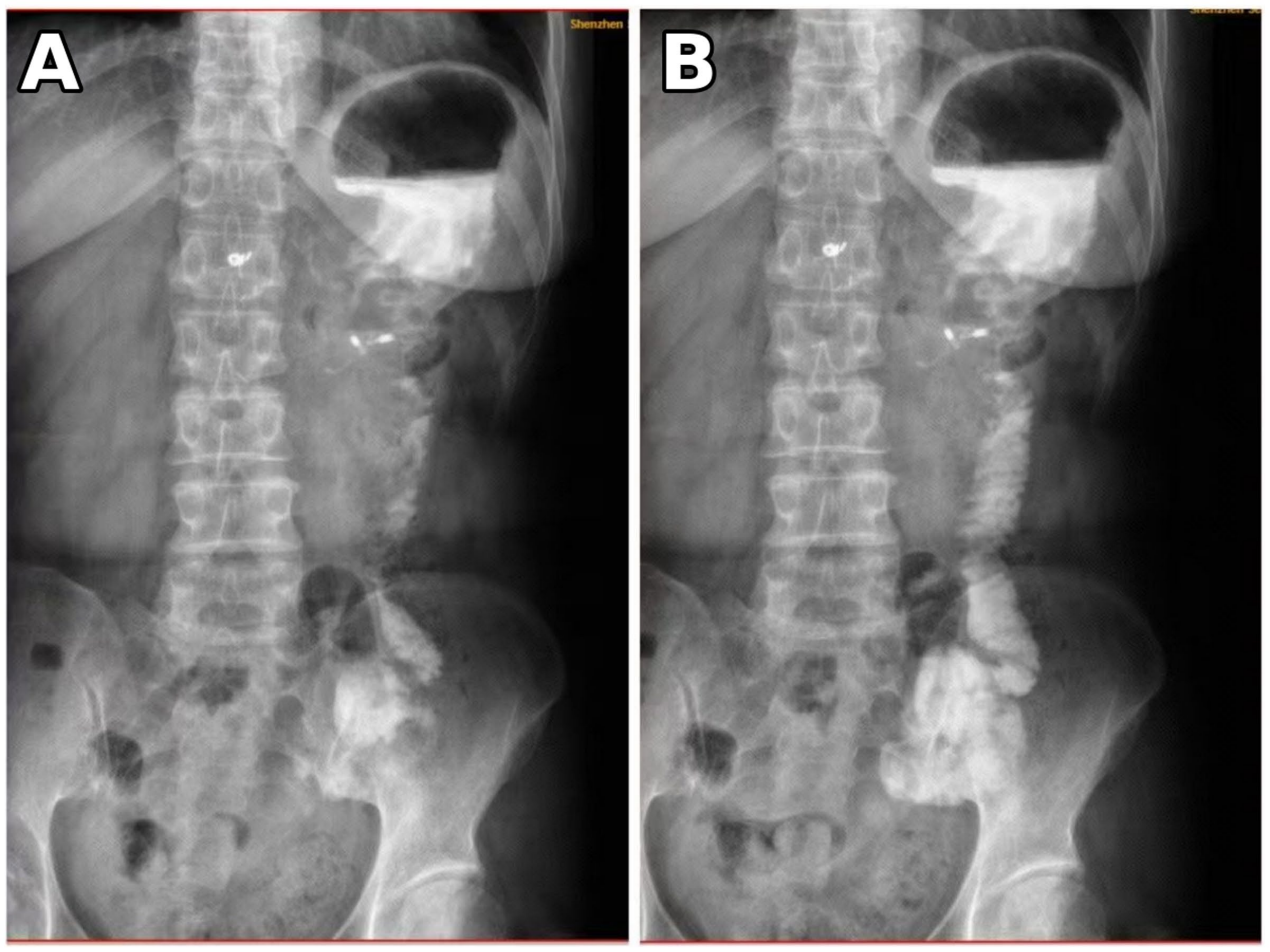
One-month post-treatment contrast study. **(A)** Radiograph obtained 30 min after oral contrast administration, showing improved passage of contrast into the efferent limb. **(B)** Radiograph obtained 3.5 h after oral contrast administration, showing smooth downstream transit without delayed gastric emptying.

**Figure 4:**
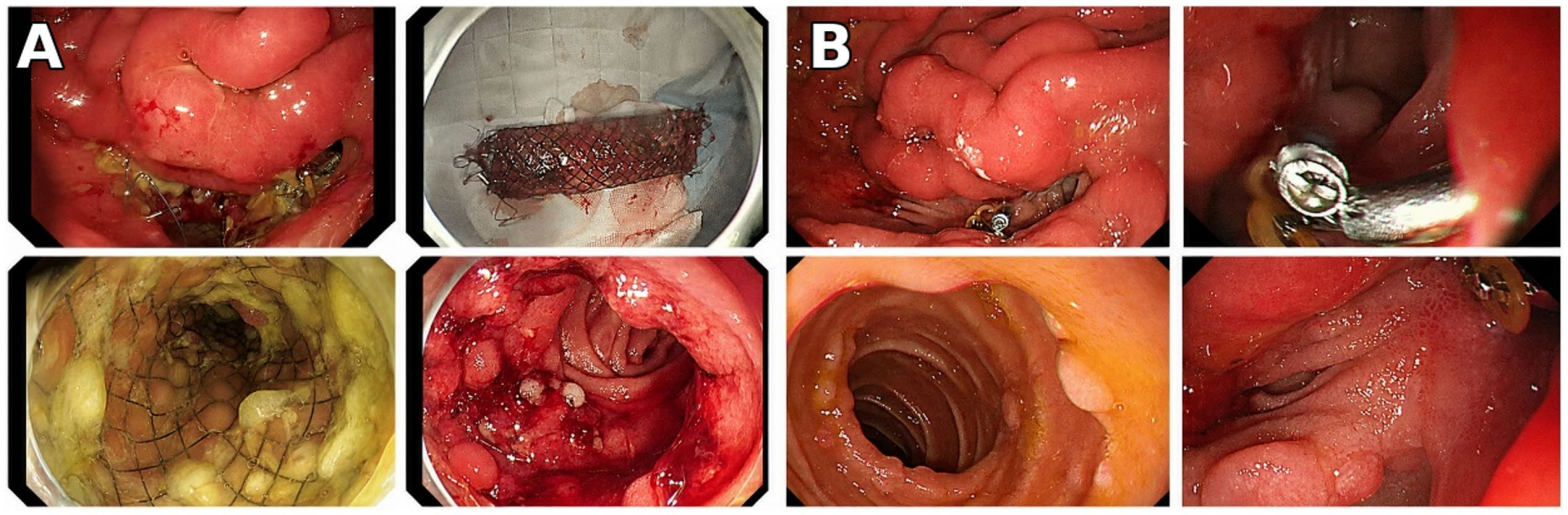
Follow-up endoscopy. **(A)** One-month follow-up endoscopy before and after stent removal. **(B)** Three-month follow-up endoscopy showing a patent efferent loop without recurrent stenosis.

At 1-month follow-up, a barium meal examination showed no delayed gastric emptying and smooth passage of contrast into the efferent limb (Figure 3). Endoscopy confirmed a well-healed anastomosis and a straight, patent efferent loop. The FCSEMS was removed endoscopically without difficulty (Figure 4A).

At 3 months, the patient reported good appetite and no further episodes of vomiting or abdominal distension. Her body weight had increased by about 5 kg compared with the time of discharge. Follow-up endoscopy again showed a widely patent efferent loop without recurrent angulation or stenosis (Figure 4B). No late adverse events related to the procedure were noted during the follow-up period.

## Discussion

ELS after PD is uncommon but can significantly affect postoperative recovery. When DGE is caused by ELS, patients may require prolonged nasogastric decompression, parenteral nutrition and extended hospitalization. Early reoperation is usually not preferred because of the risks associated with adhesions, fragile anastomoses and a generally weakened condition (1).

For benign postoperative anastomotic strictures, endoscopic therapy has become the first-line approach in many centers. EBD and temporary placement of plastic stents or FCSEMS have been shown to be effective in various settings, including hepaticojejunostomy and gastrojejunostomy after pancreatic or gastric surgery (2–4). These treatments are designed to relieve short, fibrotic narrowing through controlled radial expansion.

However, in some patients the main problem is not a short fibrotic ring, but rather a kinked or angulated loop related to postoperative adhesions or abnormal orientation. In such cases, simply inflating a balloon or placing a stent along the same axis may not fully resolve the geometric problem, and achieving a safe, stable coaxial position can be difficult (2, 4).

In the present case, endoscopic and fluoroscopic evaluation suggested that the efferent-loop obstruction was dominated by fixed angulation at the gastrojejunostomy, without a true concentric ring stricture. On the basis of postoperative imaging, endoscopic findings, and multidisciplinary discussion, the angulation was interpreted as a postoperative complication-related deformity rather than a definite technical error during reconstruction. We therefore considered that standard balloon dilation would not be ideal. Instead, we aimed to modify the geometry of the efferent limb (Table 1).

Limited ESD was used to remove a saddle-shaped mucosal ridge that appeared to accentuate the kink at the efferent-loop entrance. This step alone already widened the opening. Clip placement then provided traction and helped to straighten the efferent limb, changing its orientation relative to the gastric remnant. Finally, a temporary FCSEMS was placed to keep the lumen open and maintain the corrected geometry during the early healing phase. The patient’s symptoms resolved quickly, and imaging and endoscopy at follow-up confirmed that the efferent loop remained straight and patent after stent removal.

Endoscopic techniques using loops and clips have been reported for mucosal prolapse and related deformities causing efferent-loop syndrome, with good clinical results (6). Endoscopic stenting has also been described in cases of ELS after Billroth II gastrectomy (7). Our case differs in that the setting was early after PD, and the efferent-loop obstruction was mainly due to angulation. The combination of limited ESD, traction by clips and temporary FCSEMS provided a practical way to address both the mucosal component and the underlying geometric problem.

This report has several limitations. It is a single case from a center with experience in ESD and therapeutic endoscopy, and the approach may not be suitable for all institutions. The follow-up period was relatively short, and longer follow-up would be helpful to assess the risk of late recurrence. In addition, careful patient selection is essential. This method is more likely to be useful when imaging and endoscopy indicate a focal angulation or kink, rather than a long, fibrotic stricture or a complex multi-level obstruction.

Despite these limitations, this case suggests that a “geometric remodeling” approach, combining limited ESD, clip-based traction and temporary FCSEMS, can be a useful option in selected patients with angulation-type ELS after PD, especially when conventional balloon dilation is not feasible or is considered unsafe.

### Patient perspective

At 3 months after the intervention, the patient reported good appetite with no further episodes of nausea or vomiting, and no recurrent stenosis on follow-up endoscopy. She had gained approximately 5 kg during follow-up.

## Conclusion

We describe a case of early postoperative ELS after PD caused mainly by efferent-loop angulation. The combination of limited ESD to release a saddle-shaped mucosal ridge, clip-based traction to straighten the loop and temporary FCSEMS placement resulted in rapid symptom relief, restoration of oral intake and sustained efferent-loop patency after stent removal.

This endoscopic strategy focuses on correcting the geometry of the efferent limb rather than only dilating a presumed fibrotic stricture. It may be considered as a minimally invasive alternative to surgical revision in selected patients, provided that appropriate expertise and facilities are available. Further experience and longer-term data are needed to define the role of this approach.

## Data Availability

The original contributions presented in the study are included in the article, further inquiries can be directed to the corresponding author/s.
